# Influence of Reagents on Qualitative Indicators of Artificial Anti-Deflationary Phytocenosis on Waste from a Rare Earth Tailing Facility

**DOI:** 10.3390/toxics11070629

**Published:** 2023-07-20

**Authors:** Eugenia A. Krasavtseva, Victoria Maksimova, Dmitriy Makarov

**Affiliations:** 1 Laboratory of Nature-Inspired Technologies and Environmental Safety of the Arctic, Kola Science Centre, Russian Academy of Sciences, Fersman St., 14, 184209 Apatity, Russia; 2 Institute of North Industrial Ecology Problems, Kola Science Centre, Russian Academy of Sciences, Fersman St., 14a, 184209 Apatity, Russia

**Keywords:** sorbents, phytoavailability, phytotoxicity, *Festuca rubra* L., rare earth elements

## Abstract

This paper presents an assessment of the effect of various reagents on the qualitative indicators of anti-deflationary single-species sowing phytocenosis on enrichment waste from rare earth ores. It has been established that tailings of loparite ores are not suitable for biological reclamation due to low values of hygroscopic moisture (0.54–2.85%) and clay particles (17.6 ± 0.6%) and high content of bioavailable forms of aluminum (504 ± 14 mg/kg). Seeds of red fescue (*Festuca rubra* L.) were grown on the tailings of loparite ore enrichment with the addition of opoka (O), brucite (B), and vermiculite (V). The quality of the seed cenosis was assessed by the dry biomass of the above-ground parts of the plants and the plant height. A positive effect (one-way ANOVA followed by Tukey’s HSD test (*p* < 0.05 and *p* < 0.01)) of the considered combinations of reagents on the growth of above-ground biomass from 31.5% (V) to 70.3 (V + O), 82.4% (V + B), and 81.8% (V + O+B) and on plant height from 53.8% (V) up to 78.6 (V + O), 83.8% (V + B), and 75.4% (V + O+B) was revealed. The use of a combination of V + O and V + B reagents made it possible to significantly reduce the content of Al (by 19.0% and 52.8%), Sr (by 16.5% and 12.9%), La (by 65.2% and 40.6%), and Ce (by 66.8% and 41.9%) in the aerial part of the sowing phytocenosis compared to control. The results obtained here can become the basis for development of a combined sorption technology for the reclamation of technogenically disturbed lands.

## 1. Introduction

The development of the mining and mining industry is inevitably accompanied by pollution of environmental components by a wide range of pollutants, changes in landscapes in the area of mining mineral deposits, and waste storage from mining and processing of ores [1,2,3]. Solid mineral waste stored in open-air conditions is a source of pollution in nearby ecosystems due to dusting and the migration of water-soluble forms of pollutants [1,2,3]. Deflation from the surface of the tailings is due to a combination of such factors as climate (wind, precipitation regime, temperature), the almost complete absence of vegetation, artificially modified relief, and the granulometric composition of waste [4]. The dusting of tailings naturally leads to an increase in the concentration of solid particles in the atmospheric air, which affects the processes of ecosystem eutrophication, the formation of precipitation, and the regional climate. In addition, it increases the risk of the occurrence and development of respiratory and cardiovascular diseases in humans [5,6,7,8]. The storage of waste generated during the operation of rare earth deposits naturally causes an increase in the concentration of rare earth elements (REEs) in environmental components [9,10]. Recently, REEs have begun to be considered emerging pollutants due to their potential risk to humans and the environment [11,12,13]. With the development of the material and instrumental bases, the number of scientific studies on the pollution and distribution of REEs in water bodies [14,15], soils [16,17], and suspended particles in the urban environment increases [18,19].

Special attention to REEs is warranted due to their persistence, environmental toxicity, and ability to bioaccumulate in plants and bioconcentrate in the food chain. The inhibition of microbiological processes in soils caused by the action of REEs leads to disruption of the sustainable development of the soil system and difficulties in natural and artificial restoration of soil quality [20,21]. When entering the human body, REEs accumulate in the internal organs, bones, brain, hair, and blood, and can have a significant impact on the digestive, respiratory, reproductive, neurological, hematological, and immune systems [22,23].

Currently, there are three groups of methods for reclamation and conservation of industrial dumps: mechanical, physicochemical, and biological [24,25,26]. For short-term fixation of dusty areas of existing tailings, a physicochemical method is recommended with the aim of creating an anti-deflationary coating on the surface of the industrial dump by treating it with binders [27,28]. At decommissioned tailing dumps, a biological approach is used for the reclamation of dusty areas and dumps by exploiting plants that are resistant to the environment and its pollutants, typically with a well developed root system and large biomass.

Restoration of vegetation cover at tailing dumps by seeding anti-deflationary phytocenoses, especially in difficult climatic conditions, is a protracted and multi-stage process. In rare cases, stored enrichment waste has favorable characteristics that contribute to active self-growth in disturbed areas. More often, the soil deposited in tailings is characterized by extremely low or extremely high pH values, low water-holding capacity, a high content of heavy metals, and a limited seed bank [29]. To reduce the negative impacts of the above factors, the phytostabilization of technogenically disturbed lands can provide for the introduction of additives of various types [30,31,32].

The Murmansk region, which is part of the Arctic zone of the Russian Federation, is recognized as an environmentally sensitive territory due to a combination of intensive industrial activity and sensitive ecosystems undergoing long-term recovery [33,34,35]. The only mine in which rare earth ores are mined and processed to obtain loparite concentrate in Russia is located on the Kola Peninsula [36]. Local raw materials, namely, vermiculite, opoka, and brucite, have attracted attention as promising and inexpensive sorbents for potentially toxic elements. The use of a combination of local natural materials and native plants can provide a sustainable and effective solution to reduce the spread of pollution and restore vegetation while improving the hydrophysical and hydrochemical properties of soil. However, at present there is no evaluation of their suitability and effectiveness as stabilizing agents for the light group of rare earth elements, of which La and Ce are examples.

The aim of this study was to assess the effect of different reagents on the qualitative indicators of artificial anti-deflationary phytocenosis on the waste of a rare earth tailing dump.

## 2. Materials and Methods

### 2.1. A Brief Description of Tailings from the Enrichment of Loparite Ores

**Figure 1:**
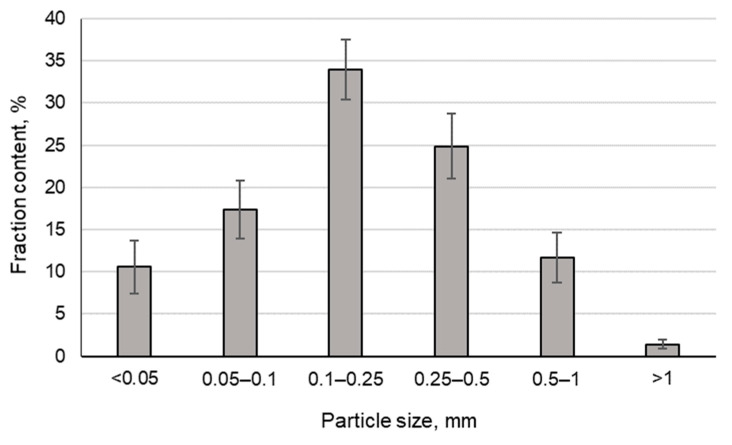
Particle size distribution in the sample from the decommissioned tailings site surface layer.

Samples of loparite ore enrichment tailings from a decommissioned tailing dump taken earlier were studied in the laboratory [37,38]. Definition of the engineering and geological characteristics was conducted by sieve, mineralogical, X-ray phase, chemical, and radionuclide analyses. The granulometric composition of the average sample of enrichment tailings taken from the tailings surface layer is shown in Figure 1. In terms of particle size distribution, the tailings are classified as fine- and medium-grained sands [39]. Nepheline, feldspars, and aegirine dominate in the mineral composition of the surface layer of the loparite ore enrichment tailings taken from the decommissioned tailings site (Table 1). Silicon and aluminum dominate in the loparite ore enrichment tailings, with a high content of sodium and iron (Table 2).

Compared to the parent rocks of regional soils [40], the ground is significantly enriched in potassium and phosphorus and depleted in calcium and magnesium. The total content of sodium and aluminum in the ground is higher than the background values as noted in the C horizon of the soils of the Murmansk region [40]. The last circumstance suggests a possible increase in the toxicity of the recultivated soil due to an increase in the lability of Na and Al at high doses of the organic matter in water-soluble forms introduced during reclamation or under the influence of the root secretions of germination plants.

### 2.2. Assessment of the Suitability of Enrichment Tailings for Biological Remediation

For this study, an assessment was made of the suitability of the loparite ore enrichment tailings from a decommissioned tailing dump for biological reclamation according to State Standard 17.5.1.03-86, “Nature Protection (SSOP), Earth, Classification of overburden and enclosing rocks for biological land reclamation” [41] based on the following indicators: granulometric composition, hygroscopic moisture, pH of water extract, and content of Al mobile forms. The content of aluminum mobile forms in the loparite ore enrichment tailings was determined by extraction with an ammonium acetate buffer solution at pH 4.8.

### 2.3. Conducting the Laboratory Experiment

Under laboratory conditions, an experiment was carried out to create a single-species seeded phytocenosis on the technosoil of the tailing dump using three types of reagents. Expanded vermiculite was used to improve the hydrophysical properties of the technosoil by increasing the moisture capacity of the substrate and loosening the surface due to the layered structure. The starting material was a type of the Kovdor deposit vermiculite with a noticeable admixture of phlogopite.

The content of the main components by wt. % was as follows: SiO_2_—30.9, MgO—27.0, Al_2_O_3_—9.6, Fe_2_O_3_—5.3, CaO—4.0, Na_2_O—3.3, K_2_O—0.9, C—0.5, H_2_O—7.7 [42]. The firing of the starting material was carried out in a modular-triggering furnace, in which the mechanism of thermal shock was implemented as the most effective method for obtaining vermiculite for 2 h at a temperature of 500 °C [43]. To reduce the content of bioavailable forms of heavy metals and aluminum, brucite and opoka were used.

Brucite, a commercial reagent (AgroMag), was chosen as a sorbent for the heavy metals and aluminum. Finely dispersed magnesium hydroxide powder (particle size < 300 µm) was produced from selectively mined natural magnesium hydroxide (brucite) by grinding and grading. The active brucite content by wt. % was as follows: MgO/Mg(OH)_2_—60.0/87.0, CaO—3.0, SiO_2_—3.0, Fe_2_O_3_—0.5 [44].

The opoka was a spent sorbent based on modified opoka (siliceous rock, produced by Alsis LLC). The sorbent was tested at the Aqua-TechFils FR-V/20 pilot water filtration unit at the Tsentralny water intake of JSC Apatityvodokanal. The task of the pilot experiments was to remove excess aluminum from the water of an underground source of drinking water; in this case, the modified opoka was used both as a filtering load and as a sorbent. The natural sorbent was extracted, crushed, activated by heat treatment at a temperature of about 1000 °C, sorted into fractions, and packaged. In finished form, it is a granular material of light orange color, with the content of the main component, SiO_2_, being up to 84%, Fe_2_O_3_ not more than 3.2%, and Al_2_O_3_, MgO, and CaO combining for 8% [45].

Loparite ore enrichment tailings were added to plastic containers (S = 0.02 m^2^, height 5 cm) in the amount of 400 g (control) and the same amount of a mixture of tailings with reagents according to the experimental scheme (experimental variants), as presented in Table 3. Seeds of red fescue were used to form a single-species seeded phytocenosis. This species was chosen for this laboratory experiment because it is native to the Kola Peninsula [46] and is widely used for restoring vegetation in technogenically disturbed areas [47,48]. It is known that *Festuca rubra* L. is tolerant to high concentrations of metals through an exclusion strategy, which confirms its recognized potential for phytostabilization [49].

First, 5 g of seeds (250 g/m^2^) were sown in each container. The seeding rate was increased compared to the traditional one [50] in order to obtain a biomass sufficient for chemical analysis. Variants of the experiment were carried out in three repetitions. At the end of 28 days of exposure, the height of the plants was measured (at least ten measurements per container) along with the weight of the aerial part after drying to an absolute dry weight. Then, the plant samples were crushed to a powdery state and transferred for chemical analysis.

### 2.4. Chemical Analysis

Samples were analyzed at the Center for Collective Use of the Institute of Problems of Industrial Ecology of the North, KSC RAS. Gross analysis of plants after decomposition of samples was carried out via the method of atomic emission spectrometry with inductively coupled plasma using an Optima 2100 DV atomic emission spectrometer. The control and quality of the analysis were ensured by the simultaneous decomposition and analysis of certified standard samples, specifically, the TR-1 mixture (GSO 8922-2007; COOMET RM 0066-2008-RU). The measurement error did not exceed 0.5% at *p* = 0.95.

### 2.5. Statistical Processing

The results were statistically processed and the arithmetic mean and standard error were calculated. The significance of differences in the variants was tested by one-way ANOVA followed by Tukey’s HSD test (*p* < 0.05 and *p* < 0.01). All statistical calculations were carried out in Microsoft Excel 2010.

## 3. Results and Discussion

### 3.1. Assessment of Suitability of Enrichment Tailings for Biological Remediation

The results of the assessment are presented in Table 4. According to the State Standard [41], soils are recognized as suitable for biological recultivation without improvement of physical properties if the content of fraction < 0.01 mm (clay) is from 10–75%. Loparite ore enrichment tailings are characterized by a very low proportion of the fraction favorable for reclamation (17.6 ± 0.6%) [42], which indicates the need for conditioning.

The hygroscopic moisture content of the loparite ore enrichment tailings is about 0.98–1.01%, which is close to the moisture content of plant wilting on technogenic lands of the European North, which is limited by values of 0.54–2.85% [51]. This allows us to conclude that the soil needs to be conditioned with water-retaining materials.

The pH of the aqueous extract of the loparite ore enrichment tailings is 8.0 ± 0.2, with an acceptable variation range of 5.5–8.4. The weakly alkaline environment of pore solutions of tailings suggests a rather low content of mobile forms of both heavy metals and rare earth elements due to the similarity of their behavior [52], which is in contrast to, for example, sulfide wastes, which result in an acidic environment [53,54]. However, under the action of root secretions of cultivated plants accompanied by a decrease in the pH of the medium, an increase in the content of bioavailable forms of microelements is to be expected [55].

According to the State Standard [41], the recognition of technogenic soil as suitable for biological reclamation is possible if the content of mobile forms of Al is not higher than an exact value of 0–30 mg/kg. The content of Al in the ammonium acetate extract is many times higher than this upper limit, at about 504 ± 14 mg/kg; according to this indicator, the ground can be considered unsuitable for reclamation. The increased content of mobile forms of aluminum is explained by the mineralogical composition of the waste, specifically, the predominance of nepheline and feldspar in the composition (Table 1) [37,38].

It is possible that this is the determining factor, as the problem of aluminum toxicity has been studied for decades [56,57,58,59]. Aluminum phytotoxicity has been reported for plants of various species, and affects the morphological and physiological parameters of plants [60,61,62,63]. Aluminum can disrupt plant metabolism by binding to macromolecules in plant tissues, inhibit photosynthetic processes and DNA synthesis, and prevent the absorption of water and nutrients [64,65,66]. Phosphorus deficiency, for which aluminum has a strong affinity, has a negative effect on the growth and development of plants [67,68]. Our preliminary assessment showed that the loparite ore enrichment tailings have unfavorable characteristics for both natural revegetation of the tailings and for biological reclamation.

### 3.2. Results of the Laboratory Experiment

#### 3.2.1. Biometric Indicators

The introduction of reagents had a generally stimulating effect on the growth of above-ground biomass. Statistically significant differences with the control variant were noted in all experimental variants (*p* < 0.05 and *p* < 0.01) (Table 5). Thus, the introduction of vermiculite increased the mass of the above-ground parts of the plants by 31.5%. A more significant increase in biomass was noted in Variants 2 (tailings + vermiculite + opoka), 3 (tailings + vermiculite + brucite), and 4 (tailings + vermiculite + opoka + brucite), amounting to 70.3, 82.4, and 81.8%, respectively.

Plant height measurements at the end of the experiment revealed a slight variability in plant height between Variants 2, 3, and 4. The average height of artificial phytocenosis plants in soil mixtures from tailings + vermiculite + opoka, tailings + vermiculite + brucite, and tailings + vermiculite + opoka + brucite increased by 78.6, 83.8, and 75.4%, respectively. The analysis of the obtained results showed that the addition of vermiculite together with the reagents had a stimulating effect on the height of plants (*p* < 0.05 and *p* < 0.01) and the growth of above-ground green biomass (*p* < 0.05) (Table 5). Statistical processing results (*p* < 0.05 and *p* < 0.01), ANOVA results, and the calculated q_tukey_ scores are presented in the Supplementary Materials.

Analysis of the statistical data processing results showed that the increase in aboveground biomass was positively affected by the addition of additives according to experimental Variants 3 (tailings + vermiculite + brucite) and 4 (tailings + vermiculite + opoka + brucite) in the absence of significant differences between them. The height of plants in comparison with the control was affected by all variants of additives, with a significant difference between Variant 3 (tailings + vermiculite + brucite) and all other experimental options.

The reaction of taller plants to the presence of high concentrations of aluminum ions in the soil solution in combination with various anions remains an incompletely understood process. It has been noted by Chauhan et al. that even a micromolar concentration of Al in the soil can cause irreversible oxidative stress resulting from the generation of reactive oxygen species and hydroxyl radicals [69]. In addition, the results of a long field experiment noted the ability of dissolved aluminum to reduce absorption of magnesium from the soil [70]. Antagonism between aluminum and calcium ions is a controversial issue, and is not always confirmed for different plants [71]. Plant tolerance to aluminum additionally depends on the genetically determined resistance mechanism developed by certain plants by stimulating the secretion of organic acids [56]. In various research works, the toxic effects of the cationic form of aluminum Al^3+^, which is characteristic of solutions with a pH less than 4 [56,69,71,72], has been widely noted. For solutions characterized by a pH of more than 5.5, on the other hand, neutrally charged Al(OH)_3_ [73] molecules predominate, which suggests a decrease in toxicity compared to the reactive cationic form.

The introduction of brucite and opoka in this case affects the soil–plant system in at least three ways. First, the ability of magnesium hydroxides and oxides to neutralize and stabilize both acidic and alkaline solutions is well known [74]. Here, it is worth considering that a significant increase in the solubility of aluminum-containing compounds is noted for pH values less than 4 and more than 8 [73]. Accordingly, the introduction of brucite into tailings, which has an alkaline reaction in our case, slightly increases the pH level and reduces the solubility of aluminum, which in turn leads to a decrease in its content in the soil solution. Second, the dissolution of brucite and exchange reactions on the surface of the mineral with the replacement of a magnesium ion by a metal ion (calcium or sodium) leads to an increase in the content of magnesium available to plants in the solution. Third, ion exchange on the surface of brucite and the flask makes it possible to reduce the sodium content (in the control, more than 10,000 mg/kg, which makes it possible to draw an analogy with weakly saline soils) in the soil solution, which is clearly noted from the results of the experiment. Such a high content of sodium in the control may have a greater effect on the inhibition of plant growth than the content of aluminum [75]. The use of red fescue in this experiment to create an artificial phytocenosis is justified in relation to the salt tolerance of this crop, along with plants such as awnless brome (*Bromus inermis*) and bluegrass meadow (*Poa praténsis*) [76], which are widely used to create artificial lawns.

#### 3.2.2. Analysis of the Content of Macro- and Microelements in the Aerial Parts of Plants

The results of the analysis of red fescue grown during the experiment and statistical processing results are shown in Table 6. Statistical processing results (*p* < 0.05 and *p* < 0.01), ANOVA results, and the calculated q_tukey_ scores are presented in the Supplementary Materials.

The results of leaf diagnostics showed that during the experiment an active accumulation of all the main nutrients (potassium, calcium, and magnesium) took place in the above-ground part of plants of the single-species phytocenosis. A rather high provision of the seed crop with these elements was noted even in the control variant, which indicates a high pool of nutrient forms available for plants in the initial technosoil. At the same time, the accumulation of nutrients in the biomass turned out to be more significant in experimental Variants 2 and 3. In addition, it is notable that the accumulation of silicon in the phytocenosis biomass in the experimental variants was up to 1.5 times greater in comparison with the control.

It should be noted that there is a decrease in the availability of Al and Na for the plants in all experimental variants. The greatest effect was achieved in Variants 2 and 3, which contributed to a decrease in the aluminum content in the red fescue from 2.1 g/kg to 1.7 g/kg and 0.99 g/kg, respectively, and sodium from 10.6 g/kg to 5.0 g/kg and 4.5 g/kg, respectively. The absorption and accumulation of aluminum, mainly in the roots of plants grown in soil contaminated with aluminum, have been reported in a number of previous works [60,62,77].

In the experimental variants with the introduction of opoka and brucite (Variants 2 and 3), a decrease in the content of strontium from 166.3 mg/kg (control) to 138.9 mg/kg and 144.7 mg/kg, respectively, was found. Remarkably, there a decrease in the content of lanthanum of 65.2% and 40.6%, respectively, and cerium of 66.8% and 41.9%, respectively, in comparison with the control variant.

An analysis of the q_tukey_ calculation results shows that for a large number of elements there is a significant difference between the experimental options and the control as well as between the different options, as the q-score exceeds the critical q level. The highest values of q_tukey_ corresponded to the introduction of additives in Variants 2 (tailings + vermiculite + opoka) and 3 (tailings + vermiculite + brucite) in comparison with the control for such elements as Al, Na, La, and Ce.

The use of reagents containing magnesium and silicon as ameliorants can be effective. Magnesium (Mg) plays an important role in the activation of many enzymes and in physiological and biochemical processes that affect plant growth [78]. As is well known, silicon can effectively increase the resistance of plants to the toxic effects of heavy metals, causing the precipitation of metals in the cell walls of root tissues and suppressing their further transport to the aerial part of the plant [79,80,81]. Silicon is additionally able to reduce the effects of salt stress through the above-mentioned mechanism of sodium accumulation in plant roots [82].

The antagonistic effects of magnesium and aluminum and resulting reduction of Al-induced oxidative stress in plants have been reported in many studies [83,84,85]. Notably, brucite can effectively remove metal ions, arsenic, and organic pollution [86,87,88,89]. The sorption properties of opoka with respect to heavy metals, phosphates, and other substances have been studied as well [90,91]. High efficiency of waste and domestic water purification by removing iron, manganese, and aluminum ions has been reported [45,92]. It is possible to obtain brucite reagents from technological sources, which are available in storage, as well as from regional water treatment enterprises upon completion of treatment [45,93].

The results of leaf diagnostics confirmed the effectiveness of adding additives in the form of a mixture of vermiculite and reagents to the loparite ore enrichment tailings to improve the nutritional regime and reduce toxicity due to the high lability of Al, thereby contributing to the stable phytostabilization of the technosoil with the aim of reducing the bioavailable concentrations of metals [94]. The use of red fescue as a seed material is justified due to its metal resistance, which largely affects the success of phytoremediation measures [95,96].

## 4. Conclusions

As a result of this study, it was found that the studied loparite ore enrichment tailings are not suitable for biological reclamation based on a number of parameters: low hygroscopic moisture (0.54–2.85%), low content of clay particles (17.6 ± 0.6%), and high content of bioavailable forms of aluminum (504 ± 14 mg/kg). To address these indicators, which limit the processes involved in overgrowth of enrichment wastes, it is proposed to use vermiculite as a moisture-retaining and conditioning agent along with opoka and brucite as sorbents of aluminum, heavy metals, and REEs.

Under laboratory conditions, an experiment was carried out to create an anti-deflationary single-species phytocenosis using different combinations of reagents. Seeds of red fescue, which is native to the study area and resistant to metal pollution, were used as seed material.

Analysis of the obtained results (one-way ANOVA and then Tukey’s HSD test) showed that the addition of vermiculite together with reagents had a stimulating effect on plant height (*p* < 0.05 and *p* < 0.01) and biomass growth (*p* < 0.05). A positive effect on the growth of above-ground biomass was revealed: from 31.5% using vermiculite alone to 70.3 using vermiculite + opoka, 82.4% using vermiculite + brucite, and 81.8% using vermiculite + opoka + brucite. Similar results were found for plant height: from 53.8% when using vermiculite alone to 78.6 using vermiculite + opoka, 83.8% using vermiculite + brucite, and 75.4% using vermiculite + opoka + brucite in comparison with the control.

The results of leaf diagnostics revealed a significant decrease in the content of Al, Sr, La, and Ce in the aerial part of the sown phytocenosis in comparison with the control. The combinations of vermiculite + opoka and vermiculite + brucite made it possible to significantly reduce the respective contents of Al by 19.0% and 52.8%, Sr by 16.5% and 12.9%, La by 65.2% and 40.6%, and Ce by 66.8% and 41.9%.

These results allow us to conclude that the reagents used in the experiment had a positive effect on the qualitative indicators of the artificial sowing phytocenosis created on the rare metal tailings dump waste in the conditions of the Russian Federation Arctic Zone. Verification of the received results in field experiment conditions remains necessary. The results obtained in this study can form a basis for the development of a combined sorption technology to be used in the reclamation of technogenically disturbed lands.

## Figures and Tables

**Table 1:** Mineral composition of the loparite ore enrichment tailings [37].

Mineral	Contain, %
Nepheline	59.17 ± 0.41
Feldspar	18.1 ± 2.03
Apatite	0.85 ± 0.2
Loparite	0.78 ± 0.15
Aegirine	19.4 ± 1.02
Diopside	0.02 ± 0.02
Sodalite	1.39 ± 0.18
Lomonosovit	0.15 ± 0.02
Lamprophyllite	0.12 ± 0.09
Eudialyte	0.02 ± 0.01

**Table 2:** Chemical composition of the loparite ore enrichment tailings.

Index	SiO_2_	TiO_2_	Al_2_O_3_	Fe_2_O_3tot._	MnO	CaO	MgO
Content, %	48.08	1.10	22.47	6.03	0.23	1.63	0.45
**Index**	**K_2_O**	**Na_2_O**	**P_2_O_5_**	**SrO**	**F**	**SO_3_**	LOI *
Content, %	4.24	13.33	0.79	0.33	0.079	0.085	1.20

Note: *—lost on ignition.

**Table 3:** Scheme of laboratory experiment.

Experience Number	Reagent	Reagent Consumption
Control	—	—
Variant 1	vermiculite	Tailings: vermiculite = 3:1 by volume
Variant 2	vermiculite + opoka	Tailings: vermiculite = 3:1 by volume + 10 g of opoka per 1 m^2^
Variant 3	vermiculite + brucite	Tailings: vermiculite = 3:1 by volume + 10 g of brucite per 1 m^2^
Variant 4	vermiculite + brucite + opoka	Tailings: vermiculite = 3:1 by volume + 5 g of brucite and opoka per 1 m^2^

**Table 4:** Assessment of the suitability of the enrichment tailings for biological remediation.

Indicator	Normative Values [41]	Actual Values
The content of fraction < 0.01 mm, %	10–75	17.6 ± 0.6
The pH value	5.5–8.4	8.0 ± 0.2
The content of mobile forms of Al, mg/kg	0–30	504 ± 14

**Table 5:** Statistical processing results: descriptive statistics.

Aboveground Biomass, g							
Groups	*n*	Sum	Mean	Variance	Interval	Minimum	Maximum
Control	3	1.12	0.37	0.01	6	4	10
Variant 1	3	1.46	0.49	0.01	4	8	12
Variant 2	3	1.89	0.63	0.00	5	9	14
Variant 3	3	2.02	0.67	0.01	4	10	14
Variant 4	3	2.02	0.67	0.01	5	9	14
**Plant height, cm**							
**Groups**	***n***	**Sum**	**Mean**	**Variance**	**Interval**	**Minimum**	**Maximum**
Control	10	65	6.5	2.72	0.23	0.25	0.48
Variant 1	10	100	10	1.33	0.24	0.36	0.6
Variant 2	10	116.1	11.61	1.53	0.14	0.56	0.7
Variant 3	10	119.5	11.95	1.25	0.19	0.57	0.76
Variant 4	10	114	11.4	2.27	0.2	0.6	0.8

**Table 6:** Content of macro- and microelements in plant samples (mg/kg).

Element	Control	Variant 1	Variant 2	Variant 3	Variant 4
K	30,344 ± 242.6	29,517.4 ± 295.7	31,496.3 ± 298.2	34,682.5 ± 354.2	27,878.8 ± 256.4
Na	10,682.5 ± 80.4	5564.5 ± 54.3	5048.7 ± 52.1	4514.8 ± 42.4	6345 ± 71.2
Ca	2588.5 ± 22.0	3175.8 ± 32.4	3574 ± 36.1	2828.6 ± 29.3	3088.3 ± 32.1
Mg	2577.4 ± 19.2	3906.1 ± 40.2	4540.9 ± 43.8	4161.9 ± 40.3	5286.5 ± 54.3
Si	10,715.9 ± 91.7	10,998.7 ± 105.8	12,557.7 ± 131.1	13,569.8 ± 142.7	15,941.4 ± 141.3
Al	2156.3 ± 20.9	1745.9 ± 14.3	1206.8 ± 11.9	995.2 ± 10.2	1576.8 ± 14.7
Cu	22.4 ± 0.5	17.5 ± 0.4	19.2 ± 0.8	21.9 ± 0.5	22.2 ± 0.9
Sr	166.3 ± 1.8	170.3 ± 1.9	138.9 ± 1.2	144.7 ± 1.8	172.3 ± 1.7
Mn	150.5 ± 1.7	164.4 ± 1.4	165.4 ± 1.5	169.4 ± 1.4	193.6 ± 2.1
Zn	69.6 ± 0.9	65.1 ± 0.9	66.8 ± 0.6	62.1 ± 0.7	62.5 ± 0.7
La	38.96 ± 0.8	33.42 ± 0.7	13.56 ± 0.5	23.12 ± 0.6	35.51 ± 0.4
Ce	92.64 ± 1.2	78.52 ± 0.9	30.77 ± 0.8	53.79 ± 0.6	85.32 ± 0.9

## Data Availability

All data are available within the article.

## Supplementary Materials

The following supporting information can be downloaded at: https://www.mdpi.com/article/10.3390/toxics11070629/s1, Table S1: The results of one-way ANOVA; Table S2: The results of Tukey’s test.
