# Supplementary material for: Influence of Reagents on Qualitative Indicators of Artificial Anti-Deflationary Phytocenosis on Waste from a Rare Earth Tailing Facility
Source: Toxics. 2023 Jul 20;11(7):629. doi: 10.3390/toxics11070629 (PMC10383936; doi:10.3390/toxics11070629)
Supplement: Supplementary file 1 [file toxics-11-00629-s001.zip › toxics-2519119-supplementary.pdf]

toxics MDPI

Supplementary Materials

Table S1. The results of one-way ANOVA

Parametr	Source of Variation	$p < 0.05$						$p < 0.01$					
		SS	df	MS	F	P-value	F critical	SS	df	MS	F	P-value	F critical
Aboveground biomass, g	Between groups	0.211627	4	0.052907	4.877689	0.019237	3.47805	0.211627	4	0.052907	4.877689	0.019237	5.994339
	Within groups	0.108467	10	0.010847				0.108467	10	0.010847			
	Total	0.320093	14					0.320093	14				
Plant height, cm	Between groups	201.7828	4	50.4457	27.70592	1.24E-11	2.578739	201.7828	4	50.4457	27.70592	1.24E-11	3.767427
	Within groups	81.934	45	1.820756				81.934	45	1.820756			
	Total	283.7168	49					283.7168	49				
K	Between groups	77831208	4	19457802	228.1556	8.89E-10	3.47805	77831208	4	19457802	228.1556	8.89E-10	5.994339
	Within groups	852830.2	10	85283.02				852830.2	10	85283.02			
	Total	78684038	14					78684038	14				
Na	Between groups	73248139	4	18312035	4820.413	2.24E-16	3.47805	73248139	4	18312035	4820.413	2.24E-16	5.994339
	Within groups	37988.52	10	3798.852				37988.52	10	3798.852			
	Total	73286127	14					73286127	14				
Ca	Between groups	1661590	4	415397.5	439.4932	3.46E-11	3.47805	1661590	4	415397.5	439.4932	3.46E-11	5.994339
	Within groups	9451.74	10	945.174				9451.74	10	945.174			
	Total	1671042	14					1671042	14				
Mg	Between groups	11885300	4	2971325	1752.849	3.51E-14	3.47805	11885300	4	2971325	1752.849	3.51E-14	5.994339
	Within groups	16951.4	10	1695.14				16951.4	10	1695.14			
	Total	11902251	14					11902251	14				
Si	Between groups	54295426	4	13573857	880.0624	1.09E-12	3.47805	54295426	4	13573857	880.0624	1.09E-12	5.994339
	Within groups	154237.4	10	15423.74				154237.4	10	15423.74			
	Total	54449663	14					54449663	14				
Al	Between groups	2493995	4	623498.9	2826.275	3.23E-15	3.47805	2493995	4	623498.9	2826.275	3.23E-15	5.994339
	Within groups	2206.08	10	220.608				2206.08	10	220.608			
	Total	2496202	14					2496202	14				
Cu	Between groups	57.156	4	14.289	33.86019	8.69E-06	3.47805	57.156	4	14.289	33.86019	8.69E-06	5.994339
	Within groups	4.22	10	0.422				4.22	10	0.422			

	Total	61.376	14					61.376	14				
	Between groups	2895.36	4	723.84	250.9847	5.55E-10	3.47805	2895.36	4	723.84	250.9847	5.55E-10	5.994339
Sr	Within groups	28.84	10	2.884				28.84	10	2.884			
	Total	2924.2	14					2924.2	14				
	Between groups	2943.336	4	735.834	273.1381	3.65E-10	3.47805	2943.336	4	735.834	273.1381	3.65E-10	5.994339
Mn	Within groups	26.94	10	2.694				26.94	10	2.694			
	Total	2970.276	14					2970.276	14				
	Between groups	116.484	4	29.121	49.19088	1.52E-06	3.47805	116.484	4	29.121	49.19088	1.52E-06	5.994339
Zn	Within groups	5.92	10	0.592				5.92	10	0.592			
	Total	122.404	14					122.404	14				
	Between groups	1302.147	4	325.5368	856.6759	1.25E-12	3.47805	1302.147	4	325.5368	856.6759	1.25E-12	5.994339
La	Within groups	3.8	10	0.38				3.8	10	0.38			
	Total	1305.947	14					1305.947	14				
	Between groups	7816.689	4	1954.172	2406.616	7.21E-15	3.47805	7816.689	4	1954.172	2406.616	7.21E-15	5.994339
Ce	Within groups	8.12	10	0.812				8.12	10	0.812			
	Total	7824.809	14					7824.809	14				

Table S2. The results of Tukey's test.

**a)** $p < 0.05$	$q_{critical\ value}$ = 4.654	$q_{critical\ value}$ = 4.018						$q_{critical\ value}$ = 4.654						
**Group pairs**	**Aboveground biomass, g**	**Plant height, cm**	**K**	**Na**	**Ca**	**Mg**	**Si**	**Al**	**Cu**	**Sr**	**Mn**	**Zn**	**La**	**Ce**
Control vs Variant 1	1.83	**8.20**	**4.90**	**143.83**	**33.09**	**55.90**	3.94	**47.86**	**13.06**	4.08	**14.67**	**10.13**	**15.57**	**27.14**
Control vs Variant 2	4.32	**11.98**	**6.83**	**158.32**	**55.52**	**82.60**	**25.69**	**110.72**	**8.53**	**27.95**	**15.72**	**6.30**	**71.37**	**118.92**
Control vs Variant 3	**4.99**	**12.77**	**25.73**	**173.32**	**13.53**	**66.66**	**39.80**	**135.40**	1.33	**22.03**	**19.94**	**16.88**	**44.51**	**74.67**
Control vs Variant 4	**4.99**	**11.48**	**14.62**	**121.89**	**28.16**	**113.97**	**72.88**	**67.58**	0.53	**6.12**	**45.48**	**15.98**	**9.69**	**14.07**
Variant 1 vs Variant 2	2.33	3.77	**11.74**	**14.49**	**22.43**	**26.71**	**21.74**	**62.87**	4.53	**32.03**	1.06	3.83	**55.80**	**91.78**
Variant 1 vs Variant 3	3.16	**4.57**	**30.63**	**29.50**	**19.56**	**10.76**	**35.86**	**87.54**	**11.73**	**26.11**	**5.28**	**6.75**	**28.94**	**47.53**
Variant 1 vs Variant 4	3.16	3.28	**9.72**	**21.93**	**4.93**	**58.07**	**68.93**	**19.72**	**12.53**	2.04	**30.81**	**5.85**	**5.87**	**13.07**
Variant 2 vs Variant 3	0.67	0.80	**18.90**	**15.00**	**41.99**	**15.94**	**14.12**	**24.68**	**7.20**	**5.92**	4.22	**10.58**	**26.86**	**44.25**
Variant 2 vs Variant 4	0.67	0.49	**21.46**	**36.43**	**27.36**	**31.37**	**47.19**	**43.15**	**8.00**	**34.07**	**29.76**	**9.68**	**61.67**	**104.85**
Variant 3 vs Variant 4	0.00	1.29	**40.35**	**51.43**	**14.63**	**47.31**	**33.08**	**67.82**	0.80	**28.15**	**25.54**	0.90	**34.81**	**60.60**
**b)** $p < 0.01$	$q_{critical\ value}$ = 6.136	$q_{critical\ value}$ = 4.874						$q_{critical\ value}$ = 6.136						
**Group pairs**	**Aboveground biomass, g**	**Plant height, cm**	**K**	**Na**	**Ca**	**Mg**	**Si**	**Al**	**Cu**	**Sr**	**Mn**	**Zn**	**La**	**Ce**
Control vs Variant 1	1.83	**8.20**	4.90	**143.83**	**33.09**	**55.90**	3.94	**47.86**	**13.06**	4.08	**14.67**	**10.13**	**15.57**	**27.14**
Control vs Variant 2	4.32	**11.98**	**6.83**	**158.32**	**55.52**	**82.60**	**25.69**	**110.72**	**8.53**	**27.95**	**15.72**	**6.30**	**71.37**	**118.92**

Control vs Variant 3	4.99	**12.77**	**25.73**	**173.32**	**13.53**	**66.66**	**39.80**	**135.40**	1.33	**22.03**	**19.94**	**16.88**	**44.51**	**74.67**
Control vs Variant 4	4.99	**11.48**	**14.62**	**121.89**	**28.16**	**113.97**	**72.88**	**67.58**	0.53	6.12	**45.48**	**15.98**	**9.69**	**14.07**
Variant 1 vs Variant 2	2.33	3.77	**11.74**	**14.49**	**22.43**	**26.71**	**21.74**	**62.87**	4.53	**32.03**	1.06	3.83	**55.80**	**91.78**
Variant 1 vs Variant 3	3.16	4.57	**30.63**	**29.50**	**19.56**	**10.76**	**35.86**	**87.54**	**11.73**	**26.11**	**5.28**	**6.75**	**28.94**	**47.53**
Variant 1 vs Variant 4	3.16	3.28	**9.72**	**21.93**	4.93	**58.07**	**68.93**	**19.72**	**12.53**	2.04	**30.81**	5.85	5.87	**13.07**
Variant 2 vs Variant 3	0.67	0.80	**18.90**	**15.00**	**41.99**	**15.94**	**14.12**	**24.68**	**7.20**	**5.92**	4.22	**10.58**	**26.86**	**44.25**
Variant 2 vs Variant 4	0.67	0.49	**21.46**	**36.43**	**27.36**	**31.37**	**47.19**	**43.15**	**8.00**	**34.07**	**29.76**	**9.68**	**61.67**	**104.85**
Variant 3 vs Variant 4	0.00	1.29	**40.35**	**51.43**	**14.63**	**47.31**	**33.08**	**67.82**	0.80	**28.15**	**25.54**	0.90	**34.81**	**60.60**

The calculated values of the q-criterion exceeding the critical values are high-lighted in bold.
